# Fitness variation in response to artificial selection for reduced cell area, cell number and wing area in natural populations of *Drosophila melanogaster*

**DOI:** 10.1186/1471-2148-7-S2-S10

**Published:** 2007-08-16

**Authors:** Vincenzo Trotta, Federico CF Calboli, Marcello Ziosi, Sandro Cavicchi

**Affiliations:** 1 Alma Mater Studiorum, Università di Bologna, Dipartimento di Biologia Evoluzionistica Sperimentale, via Selmi 3, 40126 Bologna, Italy; 2 Department of Epidemiology and Public Health, Imperial College, St Mary's Campus Norfolk Place, London W2 1PG, UK

## Abstract

**Background:**

Genetically based body size differences are naturally occurring in populations of *Drosophila melanogaster*, with bigger flies in the cold. Despite the cosmopolitan nature of body size clines in more than one *Drosophila *species, the actual selective mechanisms controlling the genetic basis of body size variation are not fully understood. In particular, it is not clear what the selective value of cell size and cell area variation exactly is. In the present work we determined variation in viability, developmental time and larval competitive ability in response to crowding at two temperatures after artificial selection for reduced cell area, cell number and wing area in four different natural populations of *D. melanogaster*.

**Results:**

No correlated effect of selection on viability or developmental time was observed among all selected populations. An increase in competitive ability in one thermal environment (18°C) under high larval crowding was observed as a correlated response to artificial selection for cell size.

**Conclusion:**

Viability and developmental time are not affected by selection for the cellular component of body size, suggesting that these traits only depend on the contingent genetic makeup of a population. The higher larval competitive ability shown by populations selected for reduced cell area seems to confirm the hypothesis that cell area mediated changes have a relationship with fitness, and might be the preferential way to change body size under specific circumstances.

## Background

Genetically based body size differences are naturally occurring in many species of the genus *Drosophila*. Such variations are usually observed in the wild as a gradual increase in size in parallel with increasing latitude, a so-called latitudinal cline [1-6], or in laboratory conditions as a response to thermal selection, with bigger flies in the cold [7-10]. Regardless of the cosmopolitan nature of body size clines in more than one *Drosophila *species, and the repeatability of laboratory thermal selection, the actual selective mechanisms controlling the genetic basis of body size variation are not fully understood.

The cellular basis of body size variation has been extensively examined to gain better understanding of body size variation from both a mechanicistic and an evolutionary standpoint. Despite the wealth of data, the study of the cellular basis of body size variation is everything but univocal and consistent. For instance, it is known that *D. melanogaster *thermal selection lines show a genetically larger size in cold adapted populations, together with a number of correlated traits that are parallel to the differences observed in flies sampled at the opposite ends of a cline [1,2,9]. Nevertheless, the cellular basis of size variation is not the same: in flies from naturally occurring clines, size variation is mostly controlled by cell number [3,11-13], while flies from thermal selection lines show size differences due to cell area variation [8,10,14]. This discrepancy could be explained if the establishment of body size clines were a two-steps mechanism, where size differences were first mediated by cell area and then by cell number [15]. Such theory is not supported by empirical observations.

The European species *D. subobscura *has recently invaded North and South America [16,17]. Two body size clines were rapidly established in the two continents, but the cellular mechanism underlying the establishment of the clines is not the same: the North American cline is based on cell area differences, the South American cline on cell number differences. The ancestral European cline is also based on cell number differences [6]. More puzzling information come from flies collected in the central area of the South American clines and kept under three different thermal selection regimes for two years. These flies show an unusual pattern of size change, mostly mediated by cell area in females and cell number in males [18].

All these observations give rise to the question of what the selective value of cell size and cell number variation exactly is. Larval competition for food can lead to reduced body size mostly due to cell mass [14,19,20]. Also laboratory adaptation to a warm thermal regime resulted not only in a smaller body size but even in an increased larval competitive ability [21]. It is known that natural populations of *Drosophila *show abundant genetic variation for body size [22] and lines of *Drosophila *artificially selected for large and small body size differ in the developmental time, with larger body size lines taking longer to develop than small or control lines [23-28].

It is possible that the mechanisms contributing to body variation through changes in cell parameters are also involved in the adaptation to new environments of *Drosophila *natural and laboratory populations. If this is actually true, one would expect a close relation between cellular components of body size and fitness. In this experiment we attempted to understand if a specific variation in a cellular component of body size is reflected in a fitness variation. In *Drosophila *the adult wing blade is composed of very flattened epidermal cells, and it has been shown that cuticular trichome density gives an estimate of wing cell area that may reflect cell size in other body regions [28,29].

In the present work we determined the fitness variation in response to artificial selection in four different natural populations of *Drosophila melanogaster*. Each population was separately selected for three different traits: reduced cell area, cell number and wing area, giving rise to three independent selection lines. In order to make general considerations about the possible relationship between variation in the cellular basis of body size and adaptation, we used different natural populations of *D. melanogaster *as independent experimental replicates. If there is a close developmental relationship between the cellular basis of body size and a fitness related trait, one would expect the artificial selection for altering one of these body size components to produce the same correlated response in a life history trait among different populations.

For all the different selection lines three fitness traits were investigated: viability, developmental time and larval competitive ability. Since the correlations between fitness traits and size in geographic populations of *Drosophila *change in intensity and sign depending on the thermal growing conditions [30-32], the association was tested both in the environment the populations were selected (25°C) and in a different thermal environment (18°C).

## Results

### Natural populations

Differences in wing area between sexes and among natural populations before selection were found, with females bigger than males (as the result of higher cell number and area) and the temperate population from Paris bigger than the temperate but warm-adapted from Georgia (USA) and the tropical ones from Brazil and Madagascar (*P *< 0.001 in both cases, data not shown). For females, the differences in wing area among natural populations were exclusively due to differences in cell number (F_3,96 _= 37.5, *P *< 0.001), since the differences in cell area were not statistically significant. For males, the differences in wing area among natural populations were mainly due to differences in cell number (F_3,93 _= 21.6, *P *< 0.001), although differences in cell area were also found (F_3,93 _= 3.19, *P *< 0.05).

### Response to selection

**Figure 1:**
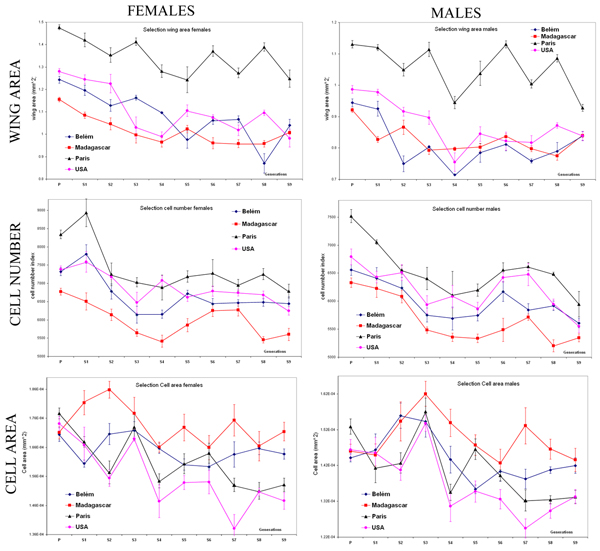
**Response to selection**. Mean values of wing area, cell area and cell number of females and males (± standard errors) of the four populations during nine generations of different selection regime.

**Figure 2:**
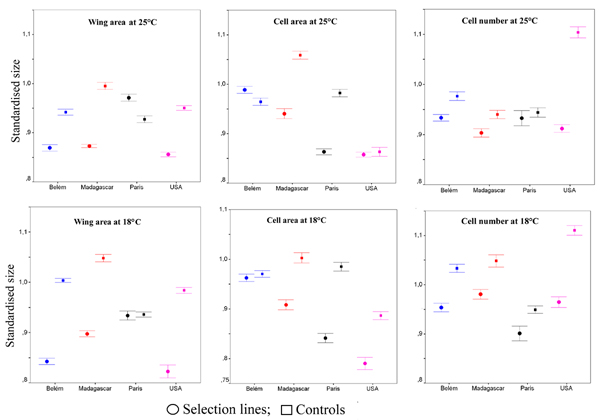
**Standardised size differences**. Standardised differences between the experimental lines and their respective base population (± standard errors) after one generation of mass-breeding for the three selection regimes. The values of males and females are pooled. Circles indicate selection lines and squares inbred control lines. Belém population is represented in blue, Madagascar in red, Paris in black and USA in violet.

Mean values of wing area, cell area and cell number of females and males of the four populations during nine generations of selection are shown in figure 1. Wing area, cell area and cell number of the populations fluctuated among generations but selection among the different populations yielded to realised heritabilities [33] (table 1) significantly greater than zero (one-tailed *t *test) for wing area (*h*^2 ^= 0.516, *P *< 0.01), cell area (*h*^2 ^= 0.527, *P *< 0.01) and cell number (*h*^2 ^= 0.598, *P *< 0.001). To correct for the differences in body size (and its cellular component) occurring among natural populations [6], we used line values divided by the respective mean values of the original outbred natural population reared in the same conditions. Figure 2 shows the standardised size values (± standard errors) of the selected populations and their respective experimental unselected control at the two temperatures after one generation of mass-breeding. Given this standardisation, it is possible to visualise body size differences between each outbred natural population (represented by the value of "1") and the derived selected and control lines. Table 2 shows the results of the mixed model ANOVAs with temperature, sex and "effect of selection" as fixed effects and population nested within "effect of selection", sex and temperature. In these ANOVAs the populations were used as independent replicates in order to test if the effect of the selection regime (i.e., the difference between the experimental controls and the selected lines) was greater than the differences among outbred populations.

**Table 1:** Realised heritability for downward selection on wing area, cell area and cell number of the four populations.

	FEMALES			MALES		
		
	Wing Area	Cell Area	Cell Number	Wing Area	Cell Area	Cell Number
Belem	0.677	0.216	0.617	0.531	0.463	0.488
Madagascar	0.646	0.643	0.426	0.206	0.415	0.787
Paris	0.362	0.616	0.639	0.288	0.527	0.527
USA	0.718	0.634	0.766	0.696	0.699	0.533

For each line, the realised heritability was calculated as the coefficient of the regression of the trait response on cumulated selection differential.

**Table 2:** Results of the mixed linear model ANOVAs (on log-transformed data) with temperature, sex and effect of selection^1 ^as fixed effects and population nested within effect of selection, sex and temperature on the standardised size values of the experimental selected populations and the inbred controls after one generation of mass-breeding for wing area, cell area and cell number.

		WING AREA		CELL AREA		CELL NUMBER	
				
*Source of variation*	*Df*	MS	*F*	MS	*F*	MS	*F*
Temperature	1	0.0046	0.38	0.024	0.91	0.071	5.18*
Sex	1	0.0077	0.63	0.0154	0.58	0.0011	0.08
Effect of selection	1	0.359	29.5***	0.2342	8.9**	0.18	13.16**
Temperature × sex	1	0.0001	0.01	0.0084	0.32	0.002	0.146
Temperature × effect of selection	1	0.0326	2.67	0.0092	0.35	0.0008	0.057
Sex × effect of selection	1	0.0044	0.36	0.0007	0.025	0.0156	1.13
Temperature × sex × effect of selection	1	0.0007	0.056	0.0019	0.07	0.002	0.15
Population nested within (effect of selection, sex and temperature)	24	0.0122	23.9***	0.0265	35.6***	0.0137	18.9***
Residuals	741/737/682	0.0005		0.0007		0.0007	

^1 ^Effect of selection: difference between inbred controls and selection lines.

*P < 0.05; **P < 0.01 ***P < 0.001; *Df*, degrees of freedom; MS, mean square; *F*, variance ratio.

The population effect was always found significant in all kinds of selection, indicating a different response to selection of natural populations. Significant differences between temperatures were found only for the lines selected for reduced cell number (*P *< 0.05); the interactions among temperature, sex and effect of selection were never found significant, indicating that both sexes responded similarly to selection and temperatures.

### Viability and developmental time

It is known that fitness traits are strongly affected by inbreeding [34-37], so the values of each fitness component of the selected lines were divided by the respective mean value of the experimental control, which is assumed to have the same level of inbreeding of the selected lines.

**Figure 3:**
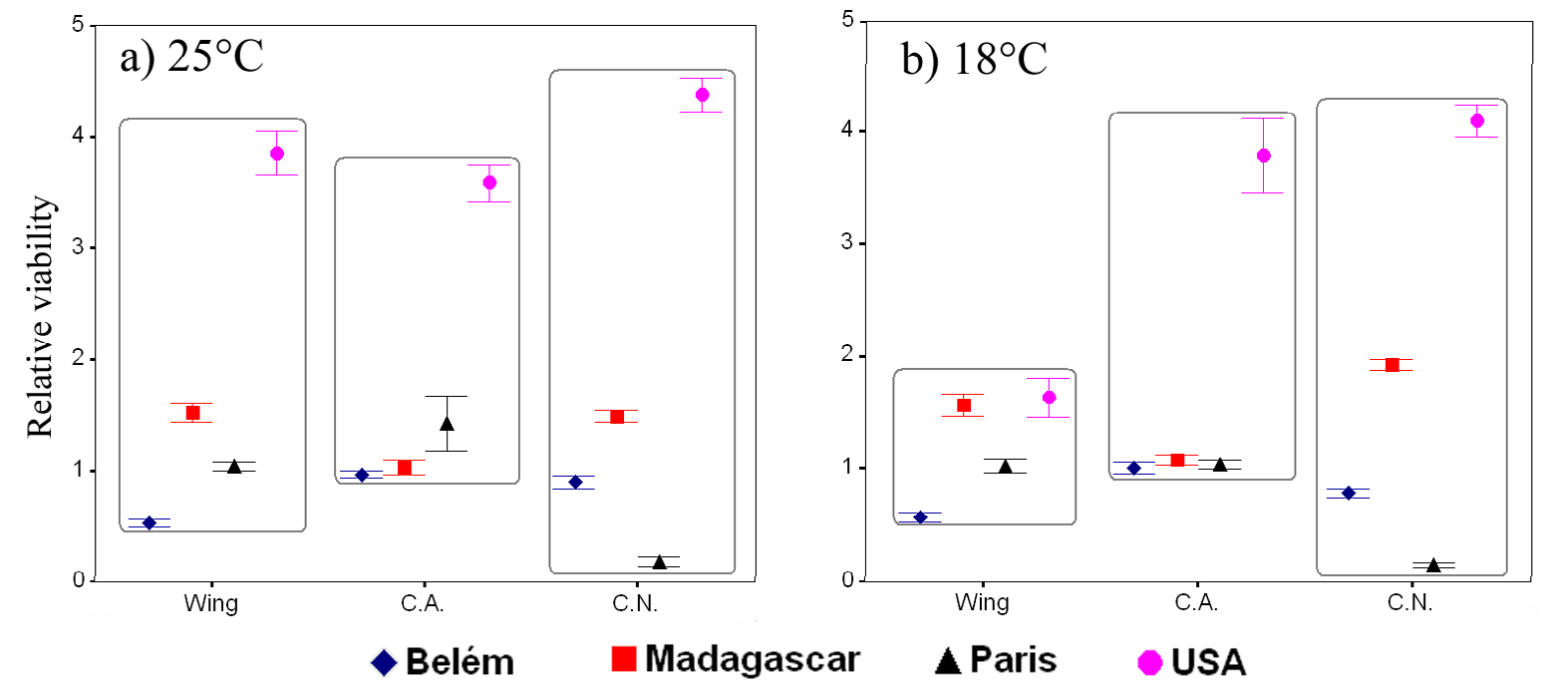
**Relative viability**. Standardised mean **v**iability (± standard error) of the selected lines at 25°C (a) and 18°C (b).

A drop in viability was recorded for flies of the Paris population selected for decreased cell number; on the contrary, an increase in viability was observed for all selection lines of the USA populations (Figure 3), possibly due to a drop in viability of the experimental control. A mixed model ANOVA was performed on the relative viability with selection and temperature as fixed effects and population nested within selection and temperature (Table 3). The population effect was significant (*P *< 0.001), while the effects of selection, temperature and their interaction were found not significant when compared with the effect of populations.

**Table 3:** Results of the mixed linear model ANOVAs on log-transformed data on the relative viability and developmental time after one unselected generation.

		Viability		Developmental time	
			
*Source of variation*	*Df*	MS	*F*	MS	*F*
Selection	2	0.377	0.239	0.0095	1.33
Temperature	1	0.0123	0.0078	0.01236	1.73
Selection × temperature	2	0.0004	0.00025	0.00078	0.109
Population (selection × temperature)	18	1.574	124 ***	0.00711	54.7 ***
Residuals	201	0.0127		0.00013	

Population is nested within selection and temperature. Standardisation: values of selected lines divided by the respective mean value of the inbred control.

***P < 0.001; *Df*, degrees of freedom; MS, mean square; *F*, variance ratio.

**Figure 4:**
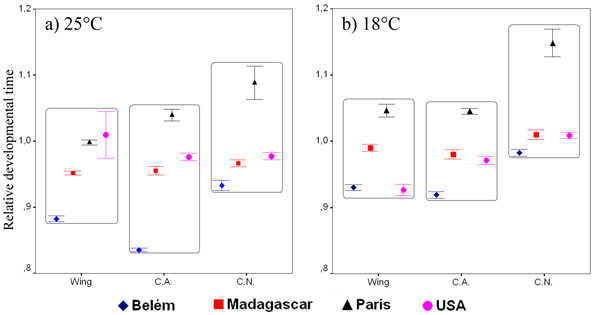
**Relative developmental time**. Standardised mean developmental time (± standard error) of the selected lines at 25°C (a) and 18°C (b).

Figure 4 shows the mean relative developmental time of the selected lines at the two experimental temperatures. The mixed model ANOVA (Table 3) gave significant differences only among populations (*P *< 0.001). As a consequence of the standardisation, differences in the duration of development between temperatures were not found; once again, differences among selection regimes were not found.

All the populations belonging to the three selection lines showed neither difference in viability nor in developmental time at 25°C and 18°C with respect to their experimental controls (*t *tests, *P *> 0.1 in all cases, data not shown).

### Larval competitive ability

For all the experimental lines (pooling the populations and the *y, w *flies), a drop in emerging adults was recorded at high larval density (i.e., 30 wild-type larvae and 90 of the *y, w *competitor stock in the same vial) respect to the optimal density (i.e., 15 wild-type and 45 *y, w*). At 25°C and optimal density, about the 58.5% of the larvae emerged as adults (on average 35 adults out of 60 larvae), whereas at high larval density only the 30% of the larvae emerged as adults (on average 36 adults out of 120 larvae). The scenario was very similar at 18°C, with the 51% of emerging adults at optimal density against the 24% at high larval density.

**Figure 5:**
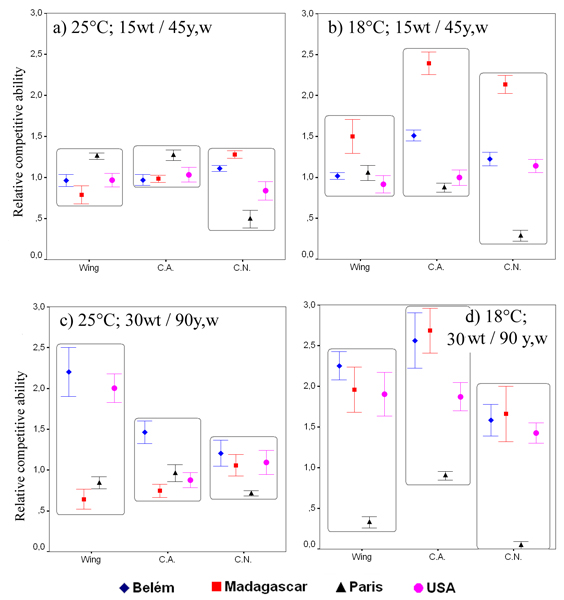
**Relative competitive ability**. Standardised mean competitive ability (± standard error) of selected lines. **a) **Relative percentage of wild-type flies from total emerging flies at 25°C and 15 wt/45 y, w density. **b) **Relative percentage of wild-type flies from total emerging flies at 18°C and 15 wt/45 y, w density. **c) **Relative percentage of wild-type flies from total emerging flies at 25°C and 30 wt/90 y, w density. **d) **Relative percentage of wild-type flies from total emerging flies at 18°C and 30 wt/90 y, w density.

As for viability and developmental time, figure 5 shows the mean values (± standard errors) of the ratio between selected lines and the respective experimental controls in the proportion of "wild-type" experimental flies surviving to adulthood respect to the total flies emerged in each vial, separated for temperature and larval density.

Two mixed model ANOVAs were performed for the two larval densities separately (table 4). Significant differences among populations were found (*P *< 0.001 in both cases), while differences among selection regimes or the interaction "selection by temperature" were not found significant. At high larval density, the temperature effect was greater (even if not significant) than the population effect, indicating that the relative performance of the lines selected for reduced wing size components was slightly better at 18°C than 25°C under the more competitive conditions.

**Table 4:** Results of the mixed linear model ANOVAs on log-transformed data on the relative percentage of wild-type flies from total emerging flies at the two larval densities.

	15 *wt*/45 *y, w*			30 *wt*/90 *y, w*		
		
*Source of variation*	*Df*	MS	*F*	*Df*	MS	*F*
Selection	2	0.2	0.58	2	0.089	0.187
Temperature	1	0.209	0.6	1	1.24	2.6
Selection × temperature	2	0.027	0.078	2	0.329	0.69
Population (selection × temperature)	18	0.346	15.7 ***	18	0.476	13.4 ***
Residuals	216	0.022		209	0.0354	

Population is nested within selection and temperature. Standardisation: values of selected lines divided by the respective mean value of the inbred control.

***P < 0.001; *Df*, degrees of freedom; MS, mean square; *F*, variance ratio.

Only in one case a significant difference between populations and their respective experimental controls was found. At high larval density and at the temperature of 18°C, the lines selected for reduced cell area competed better than their inbred controls (*t *test, *P *= 0.045, fig. 5d). Figure 5d also shows that the three "warm adapted" selection lines relatively improved (compared to their unselected controls) their competitive ability against the *yellow white *strain at 18°C and high larval density, but the Paris selection lines did not.

To test if the increased larval competitive ability at 18°C were due to a specific effect of selection for decreased cell area (genetic effect) or to a phenotypic effect of small cell area (directly produced by selection on this trait or as a response correlated to selection on cell number or wing area), we correlated the cell size over all kinds of selection and populations (n = 12) with competitive ability, obtaining a not significant correlation (*r*_(10) _= 0.168, *P *= 0.6).

## Discussion

*D. melanogaster *is known to exhibit numerous genetic differences between populations with some life history traits indicative of temperature selection in tropical and temperate populations (body size, duration of development and offspring production) [31]. The control of body size depends on the integration of various genetic and environmental causes that operate through complex molecular and physiological mechanisms [20,38,39].

In *Drosophila*, wing area is positively correlated with body size as a whole [14,22,40,41]. Differences in wing size among flies from natural populations, from thermal selection lines, from artificial selection, from different temperatures or different larval density are caused by variations in cell area, cell number or both.

In the present study, different geographic populations of *D. melanogaster *with different past selective histories were used as independent replicates for three selection experiments. The response to selection for reduced cell area, cell number and wing area confirmed the existence of independent genetic variance for those traits [10,14,11]. Artificial selection altered the mechanisms that regulate cell growth and proliferation and our aim was to test whether these developmental changes had any measurable evolutionary consequences.

Within each kind of selection, viability did not change between the selected populations and their relative experimental controls. Moreover, viability seemed to be independent of the selection regime and temperature; differences were found only among selected populations (table 2): given the standardisation for the respective experimental control, one possible explanation is that the relationship between viability and wing traits, if any, is population dependent and not trait dependent.

Developmental time may be important in nature; in *Drosophila*, a rapid development reduces fly age at the first breeding and so would be at a premium. Our results showed that selection for reduced wing size or for its cellular components was not correlated with developmental time to adulthood. A similar result was found by Partridge *et al. *[28]: flies selected for reduced body size achieved their smaller size not by modifying their developmental time, but reducing their growth rate from an early stage. If reducing growth in *D. melanogaster *has no effect on developmental time, shortening developmental time has instead an effect on growth. Direct selection for a shorter developmental time led to a smaller adult size [42,43]. A small body size can be achieved by reducing developmental time (keeping growth efficiency constant) or by reducing growth efficiency (keeping developmental time constant). On these bases, developmental time and growth efficiency can be considered two independent traits, that produce different correlated response when subjected to artificial selection.

In this work we show that individuals selected for small cell area have a larval competitive advantage at 18°C, particularly evident for the three "warm adapted" selection lines. Larval crowding has a great impact on fitness through its effect on growth and survival of larvae; competition for food also leads to reduced adult body size (mostly due to cell size reduction) [19,23]. Thermal evolution in the lab led to smaller flies in hot environments through variation in cell area [8,10,14]. Larval competitive success of thermal selected lines was found higher at the rearing temperature corresponding to their evolutionary history [21]. However, when the larval density was increased, the warm-adapted lines performed equally to the cold-adapted lines when tested at low rearing temperature [21,44]. Moreover, an analysis of geographic variation in larval competitive ability performed on natural populations from an Australian north-south cline failed to support those findings conclusively [45].

On these bases it seems worthwhile to suggest that in many laboratory thermal selection experiments at high temperatures, where a higher larval competition is expected [18,46,47], an increase in pre-adult competitive ability could be the selective driving force in reducing body size through a decrease in cell area, probably by reducing the feeding associated costs [48]. On the other hand, in nature, larval adaptation to crowded conditions could not be the only driving force involved in the evolution of body size observed among natural populations of *Drosophila*, where size differences are mainly associated to the number of cells.

The larval competitive advantage, only evident at 18°C, could be explained considering the opposite behaviour of cell area in response to the effect of high larval crowding and low temperature. If the effect of low temperature leads to an increase of body size, the competition for food leads to smaller flies (both effects are mostly mediated by cell area variation). However, only larvae that reach the "minimum viable weight" [49-52,20] survive to adult, since the major selective factor in this occasion is the increased larval crowding. When the two effects occur simultaneously, it is possible that selection for reducing cell area produce a genetic reduction of the "minimum viable weight", opposite to the physiological increase due to the effect of temperature, which allows a higher survival of starved larvae.

## Conclusion

The evolutionary significance of a trait is argued by its impact on fitness, at least under specific environmental conditions. In this work we failed to detect any correlation between the cellular components of body size, after selection in various unrelated populations, and two fitness components: viability and developmental time. These two traits change in selected lines in a way which seems dependent on the contingent genetic makeup of a population with no clear dependence on the kind of selection.

More interesting, we found that flies selected for smaller cells showed an increase in larval competitive ability, compared to their unselected controls, at a low temperature, especially for the warm adapted populations. The temperate Paris selection lines appear to be relatively unable to compete; nevertheless, looking at the differences between the three selection regimes, even for the Paris population, the line selected for reduced cell area has an increase in larval competitive ability respect to the other two lines selected for reduced wing area or cell number. In spite of the differences between warm and cold adapted populations, our results strongly suggest that the relationship between wing traits and fitness is trait dependent and not only population dependent [48,53,54]. This observation supports the hypothesis that changes in cell area might be the preferential way to move body size under specific circumstances, thermal selection (experiments) included.

Our experiment suggests that the relationship between the cellular basis of body size and fitness is more complex than previously thought. The developmental mechanisms responsible for body size evolution through changes in cell area and/or cell number might take a more efficient route in some cases – or even in all cases – but at the same time the genetic variability present in a population might ultimately be the way organisms respond to selection.

## Methods

### Populations

Four different natural populations of *Drosophila melanogaster *were used. The first is a temperate population that comes from Draveil, near Paris (48°44'N, collected in 2002); the second temperate but warm adapted population comes from Athens, Georgia-USA (33°57'N, collected in 2002). The tropical populations come from Belém, Brazil (1°27'S, collected in 2002) and from Mananara, Madagascar (16°10'S, collected in 2000). All strains (kindly provided by J. R. David) were founded starting from 10–15 pairs of wild-collected flies and kept in mass culture on standard medium and controlled density at 20°C until 2004 when the experiment started.

### Wing area, cell area and cell number measurement

**Figure 6:**
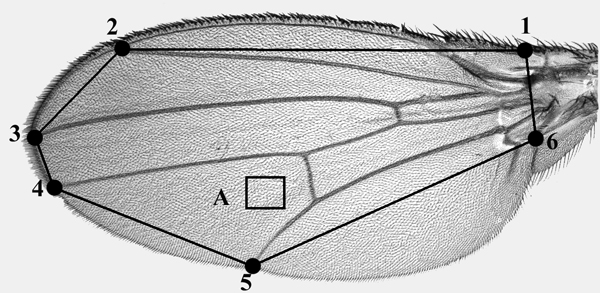
***Drosophila melanogaster *wing**. The black outline superimposed on the wing joins the six points (1–6) used to determine wing area. The box (A) indicates the standard region used for trichome counting to estimate average cell area. On wings of different size, the region was chosen corresponding to the equivalent location with respect to veins and wing margin.

Left wings of females and males were dissected, dehydrated in ethanol and mounted on glasses in lactic acid/ethanol (6:5). Wing images were captured using a Zeiss optical microscope mounting an Axiocam digital camera. The 50 × optical magnification was subsequently enlarged through a 2 × digital zoom. The outline of each wing was traced starting at the alar-costal break (Fig. 6) and the area was taken using Image Pro Plus software [55], on the basis of the number of pixels included in it. The estimate of the cellular components of body size is the same described in Santos *et al. *[18]. An image of the left wing was taken at 40 × 10 magnification, and a sampling square of 11.55 × 10^-3 ^mm^2 ^was selected in the area of the wing proximal to the posterior cross vein (Fig. 1). Trichome counting followed a standard protocol; namely, the sampling area was visually inspected and the trichomes whose roots were within the selected square were marked with a black dot. Further manipulation provided a final image showing only the dots, which were counted using the ImageJ software [56]. Cell area was then estimated as 11.55 × 10^-3 ^mm^2^/dot number. Because cell area is variable across the wing blade, a total cell number index was estimated as wing area (mm^2^)/cell area (mm^2^).

### Selection lines

To generate the parents for the selected lines (and to avoid maternal effects), flies of each outbred geographical population were allowed to oviposit for a day at the constant temperature of 25°C and at an optimal density (about 100 individuals in 60 ml vials containing 10 ml of food) for one generation. For each population, 25 virgin females were collected and randomly paired with single males. Each pair was placed in a separate numbered vial with standard food, allowed to mate and oviposit for 6 days, changing the vial every two days in order to avoid crowding effects (that reduce body size and its cellular component) due to possible differences in fecundity and larval viability among pairs. The pair was then killed, the left wing was removed, mounted and scored for wing area, cell area and cell number. The pairs with the smallest value of these traits were chosen as the parents for the next generation.

A control line for each population was also founded randomly choosing a vial among the 25 used. This protocol was used for all the 4 populations to start a control and three independent selection lines.

Subsequently, the adult flies emerged from the selected vial were collected as virgins and stored at 18°C. After few days, up to 12 females were again randomly paired with single males and allowed to mate and oviposit for 6 days, changing the vial every two days. From the fertile pairs, the left wings of both male and female were removed and measured, so in each generation only one pair of flies out of the 5–10 measured was used as parents for the next generation in each line. The lines were selected for 9 generations, while the control line was maintained in parallel to each selected line. At each generation the control line was seeded with the same protocol of the selection experiment, but these flies were a randomly chosen subset of the control. All the selection experiment was carried out at 25°C.

### Experimental procedures

After nine generations of artificial selection, flies were collected and maintained without selection for one generation. 10 pairs were placed in a 60 ml vials containing 10 ml of food and were allowed to oviposit for 2 days at the constant temperature of 25°C. The emerging flies of the selected and control lines were transferred in bottles with an egg laying dish containing apple juice medium smeared with abundant yeast. To collect synchronised eggs, females were allowed to oviposit for two days at 25°C, changing the egg laying dishes two times a day; at the third day the dishes were changed three times every 2 h, then the egg collection started. 30 eggs were counted and transferred in 30 ml vials containing 5 ml of food (optimal density). Twenty vials (when available) for each line were maintained until adult emergence at the different constant temperatures of 25°C and 18°C (ten vials for each temperature). Individuals from this group of vials were used to measure viability, developmental time to adulthood and wing size components. In order to have a better understanding of size variation due to possible inbreeding effects, ten vials of the original non selected populations were left at 18 and 25°C in the same conditions of the selected ones.

To examine fitness during the pre-adult period, a larval competition experiment was established for the selected lines of each population and for the experimental controls. To detect the effects on "egg to adult" survival rates, two types of density treatments were chosen: the first one consisted of 15 experimental (selected and control lines) first instar larvae with 45 first instar larvae of a *yellow, white *stock (kept for several years at 18°C) in the same vial with 5 ml of food; the second one consisted of 30 experimental first instar larvae with 90 of a *yellow, white *stock, with the same level of food as the first treatment. The larvae used in this experiment were obtained from the egg laying dishes changed before collecting the synchronised eggs for the detection of developmental time. The same protocol was used for the competitor stock. The competition assays were repeated at two temperatures: 25°C and 18°C. For each selected and control line, 10 vials (when available) were seeded per each combination of density by temperature.

Competitive ability was estimated as the percentage of wild-type flies from the total emerging flies within vial. This procedure has the effect of increasing larval crowding and uses a competitor stock as a yardstick against which to measure the competitive ability of other strains, giving a more sensitive index of competitive ability than pure cultures do [57,58].

### Developmental time and viability

Developmental time was measured as the days elapsed between egg laying and adult emergence. Flies were collected three times a day at both experimental temperatures. These flies were also used to investigate viability, wing area, cell area and cell number. The developmental time was calculated as the grand sum of the number of emerged adults (n_i_) multiplied by the time at which they eclosed (t_i_, in days), all divided by the total number of emerged flies (N), that is = (Σ n_i _* t_i_)/N. Viability was estimated as the percentage of flies emerged from the counted eggs.

All the analyses in this work were performed with R 2.2.0 [59]. The ANOVAs on the response to selection, viability, developmental time and larval competitive ability were done on log-transformed data.

## Competing interests

The authors declare that they have no competing interests.

## Authors' contributions

VT designed and performed the study, carried out the statistical analyses and drafted the manuscript. FCFC designed the study and carried out the statistical analyses. MZ made substantial contributions to the final manuscript. SC coordinated the experiment and drafted the final manuscript. All authors read and approved the final manuscript.
